# Ipsilateral Hemiparesis in Lateral Medullary Syndrome: A Case Report on Opalski’s Syndrome

**DOI:** 10.7759/cureus.76105

**Published:** 2024-12-20

**Authors:** Amit Agarwal, Pavni Agrawal, Kunal Suri, Diki P Theengh, Himanshu Kaushal

**Affiliations:** 1 Neurology, Mahatma Gandhi Medical College and Hospital, Jaipur, Jaipur, IND

**Keywords:** hemianesthesia, horner’s syndrome, ipsilateral ataxia, ipsilateral hemiparesis, lateral medullary syndrome (wallenberg syndrome), nystagmus, opalski's syndrome, vertebral artery thrombosis, vertigo, vomiting

## Abstract

Lateral medullary syndrome (LMS) is a neurological disorder usually presenting as loss of pain and thermal sensation over the ipsilateral face and contralateral half of the body, ipsilateral limb ataxia, Horner’s syndrome, dysphagia, nystagmus, hiccups among other symptoms but never with limb weakness. In the present case, the patient presented with ipsilateral hemiparesis, which can be attributed to the extension of the infarct caudally beyond the pyramidal decussation, affecting the corticospinal fibers in the upper cervical cord, a variant of LMS, known as Opalski syndrome (OS).

## Introduction

Lateral medullary or Wallenberg syndrome is a well-documented vertebrobasilar vascular syndrome. Identification of lateral medullary syndrome (LMS) is easy, as it affects a limited area of the brainstem with a distinct presentation and a characteristic blood supply [1,2]. We present a case of Opalski syndrome, a rare form of Wallenberg syndrome, in which lateral medullary syndrome (LMS) is accompanied by ipsilateral hemiparesis, which is not a typical characteristic presentation of LMS. This report underscores how variations in the involvement of the lateral medulla can lead to a range of clinical manifestations.

## Case presentation

**Figure 1:**
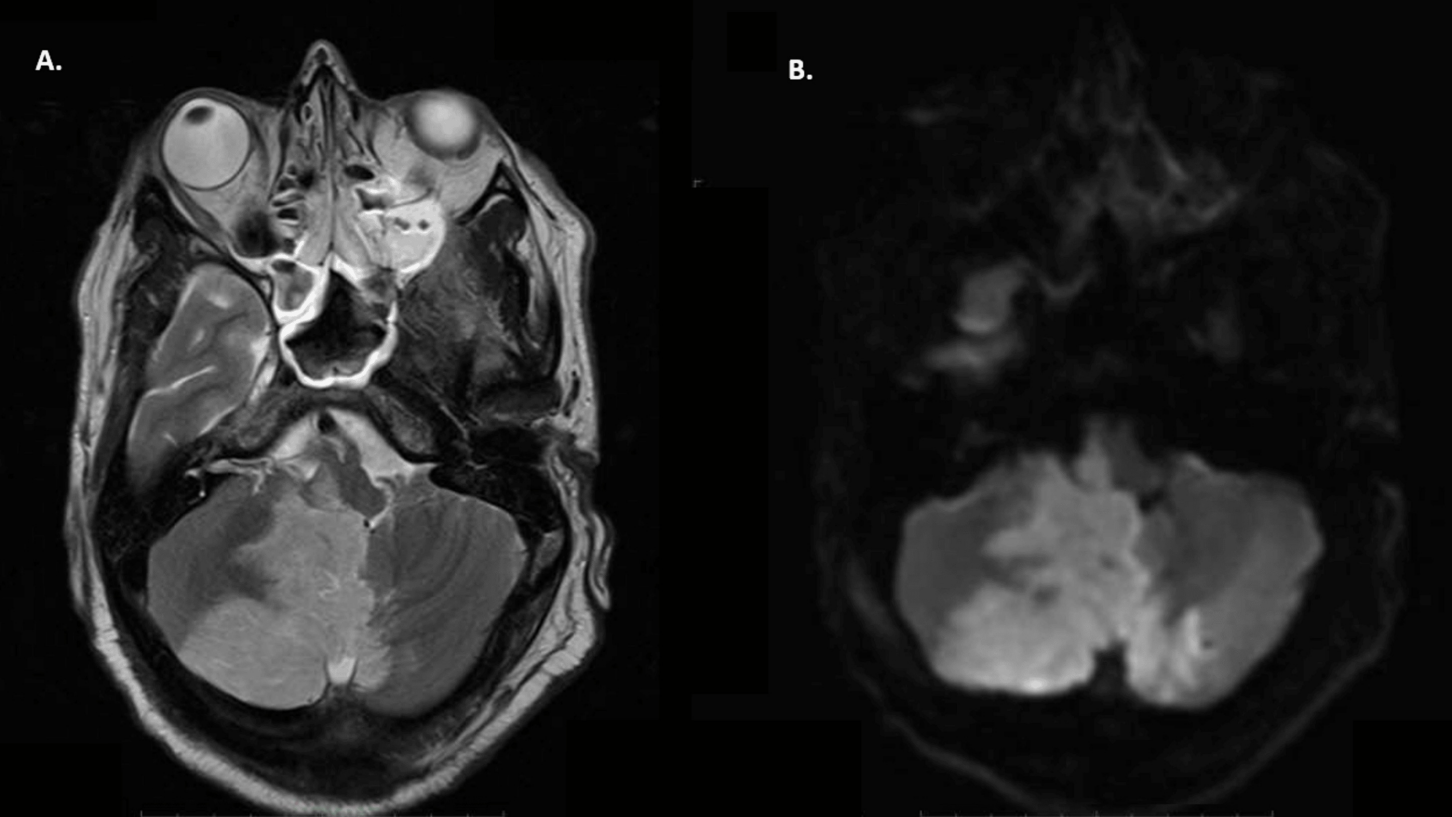
MRI Brain (A): T2 axial section at the level of medulla showing hyperintense signal in right lateral medulla along with right cerebellar hemisphere; (B) DWI axial section showing areas of diffusion restriction in the same territory suggestive of acute infarct

**Figure 2:**
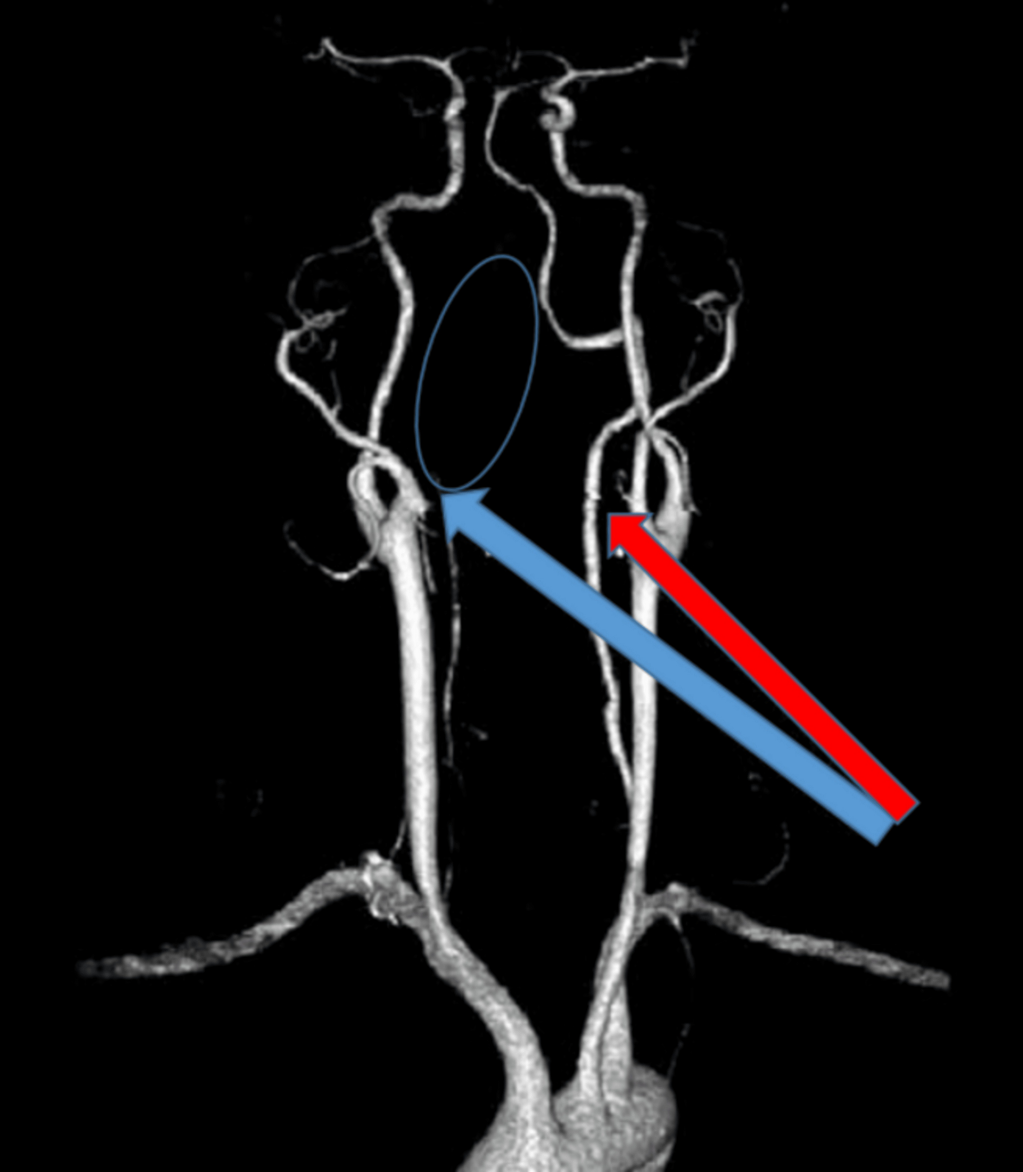
CT angiography of brain and neck vessels showing a hypoplastic right vertebral artery (blue arrow) and thrombotic occlusion of the intracranial segment of the right vertebral artery (blue circle) and the left vertebral artery (red arrow)

A 55-year-old right-handed male presented to the neurology department with sudden-onset vertigo for three days, which was followed by episodes of vomiting. The patient developed weakness on the right side of the body along with numbness over the left side of the body, dysarthria, and gait abnormality in the form of swaying to the right side. Over a span of two days, he developed dysphagia, became drowsy, and was intubated in another hospital in view of his poor Glasgow Coma Scale (GCS) score. On examination, his blood pressure was 200/120 mm of Hg, heart rate was 84 beats per minute, and oxygen saturation was 96% on a mechanical ventilator. His GCS was E4VTM6, and he was responding to commands by raising his left arm and leg. Power in his right upper limb and lower limb was 2/5, and his deep tendon reflexes were Medical Research Council (MRC) grade 2+. Although the muscle tone and bulk were normal, he had extensor plantar response on the right side. He had hypoesthesia over the left side of the body. Magnetic resonance imaging (MRI) of the brain showed acute infarcts in bilateral cerebellar hemispheres (right>left), vermis, and the right posterolateral aspect of the right medulla oblongata (Figure 1). Usually in a patient with right medullary infarct, the presentation of LMS is with difficulty in swallowing, change in speech, nystagmus, vertigo, ipsilateral Horner’s syndrome, ipsilateral facial numbness and contralateral limb numbness, ataxia, but without any weakness of the limbs. Our patient presented with right-sided limb weakness along with other features. The diagnosis of OS was made given the classical clinical presentation and examination findings. Computed tomography (CT) angiography of brain and neck vessels (Figure 2) was done which showed a hypoplastic right vertebral artery with a complete block of the right vertebral artery in the intracranial segment with a non-opacified posterior inferior cerebellar artery on the right side. During the course of his stay, he developed Horner’s syndrome of the right side in the form of miosis and ptosis, along with intractable hiccups. He later developed respiratory depression such that multiple efforts to wean him off were unsuccessful. The patient developed ventilator-associated pneumonia later on during the course of the stay, and his attendants refused further treatment and left against medical advice with regard to poor prognosis.

## Discussion

Opalski syndrome presents as weakness on the same side of the vascular lesion along with other features of LMS, making it a rare variant of Wallenberg syndrome. First described in 1946 as ipsilateral weakness with hyperreflexia and features of LMS and confirmed by comprehensive neurological examination and MRI findings, the cause of weakness in OS is due to the extension of the infarct caudally beyond the pyramidal decussation to involve the corticospinal fibers of the upper cervical spinal cord [1-3]. Localization of LMS as described earlier is straightforward, as along with having a distinctive presentation and specific blood supply, findings of MRI and CT angiography aid in the diagnosis. However, it can pose difficulty for localization to those who are not well-versed with the variants of LMS. Mechanisms causing OS are varied and could be due to occlusion caused by atherosclerosis of the vertebral artery or the posterior inferior cerebellar artery. Ultimately, the medullary penetrating arteries are involved, which supply the pyramidal fibers post-decussation, which seems to be the cause in our case. Another cause of OS is vertebral arterial dissection. The cardio-embolic phenomenon is, however, a rare cause. Hermann DM et al. (2009) described alternating and dissociative sensorimotor symptoms in two cases characteristic of OS [4]. Yi Yang et al. (2023) described a case of a 32-year-old male with right-sided hemiplegia, contralateral numbness of the limbs, ipsilateral numbness of the face, and ataxia, who was diagnosed as having OS. MR angiography showed right vertebral arterial dissection, and he was started on dual antiplatelet therapy and recovered completely in three months [5].

A comprehensive systemic analysis by Rukerd MRZ et al. (2024) revealed a clear male predominance, with males making up 76.60% of cases. Common risk factors identified were hypertension (63.54%), smoking (32.39%), diabetes (32.29%), and alcohol use (22.91%). OS has been reported in 22 countries spanning 5 continents, with the majority of cases occurring in Asia (77.08%) [6]. The early symptoms often resemble those of LMS, and the presence of ipsilateral hemiparesis complicates the diagnosis. Accurate identification frequently depends on advanced neuroimaging and a thorough understanding of the variants of Wallenberg syndrome. Though the syndrome has an overall mortality rate of 4.16%, there have been no recorded deaths since 2014, indicating progress in treatment and management strategies. Understanding OS is challenging for rehabilitation personnel as they should be able to differentiate ipsilateral ataxia and hemiplegia from OS, so that appropriate assessment, goal setting, and rehabilitation treatment can be provided [7].

## Conclusions

LMS can manifest with diverse clinical presentations, and when the typical pattern is absent, it may suggest atypical variants, such as Opalski syndrome (OS), where ipsilateral hemiparesis is a hallmark feature. Effective management hinges on early recognition of symptoms, precise risk factor management, and the application of advanced neuroimaging techniques to optimize patient outcomes. It is crucial for clinicians to stay informed about OS to enhance diagnostic accuracy and customize treatment strategies for better patient care.
